# A Case Report on the Trilogy of Yellow Nail Syndrome: Yellow Nails, Pleural Effusion, and Lymphedema

**DOI:** 10.7759/cureus.66690

**Published:** 2024-08-12

**Authors:** Bipin Adhikari, Biplab Adhikari, Hari G Madichetty, Alina Dhital, Ashok Kumar Kanugula

**Affiliations:** 1 Internal Medicine, Wellstar Spalding Medical Center, Griffin, USA; 2 Infectious Diseases, University of Louisville School of Medicine, Louisville, USA; 3 Pulmonology and Critical Care Medicine, Wellstar Spalding Medical Center, Griffin, USA; 4 Internal Medicine, Essen Health Care, New York, USA

**Keywords:** chronic cough, bronchiectasis, case report, lymphedema, pleural effusion, yellow nail syndrome

## Abstract

Yellow nail syndrome is a rare medical syndrome characterized by the combination of a triad of yellow nails, recurrent pulmonary manifestations, and lymphedema. All three features of the triad may not be present synchronously. The diagnosis is made clinically once other causes have been excluded. Typically, it occurs in individuals who are 50 years old and above. We report a case of yellow nail syndrome in a 62-year-old male who presented with recurrent episodes of difficulty breathing due to pleural effusion. Further examination revealed pitting edema of the bilateral lower extremities. In the later encounter, his nail was found to be yellowish. Excluding other diagnoses like heart failure, fungal infections, autoimmune diseases, and lung cancer, with a typical triad, a diagnosis of yellow nail syndrome was made. He was managed with pleural fluid tapping for pleural effusion, compression stockings for leg edema, and vitamin E for nail changes. The study also intends to highlight current treatment options and alert physicians of this syndrome with such typical findings.

## Introduction

Yellow nail syndrome is a complex medical condition with a triad of yellow discoloration of nails, pulmonary involvement (chronic cough, bronchiectasis, pleural effusion), and lymphedema. The prevalence of yellow nail syndrome is fewer than one in a million with no more than 400 cases found in the literature [1]. It is believed to be an acquired entity possibly due to lymphatic disturbance both structurally and functionally or possibly due to the increased permeability of microvascular architecture. As all three symptoms of the triad are present in only one-third of the cases, diagnosis usually requires only two of the three components [2]. Early diagnosis is important as it may be the initial manifestation of an underlying malignancy [3].

We present the case of a 62-year-old male with recurrent episodes of difficulty breathing secondary to bronchiectasis who had examination findings of yellow-colored nails of both hands and feet along with pitting edema of the bilateral lower limbs. He was diagnosed with yellow nail syndrome. He was managed with compression stockings, pleural fluid tapping, and vitamin E and has been kept on regular follow-ups to look for the resolution of symptoms.

## Case presentation

**Figure 1:**
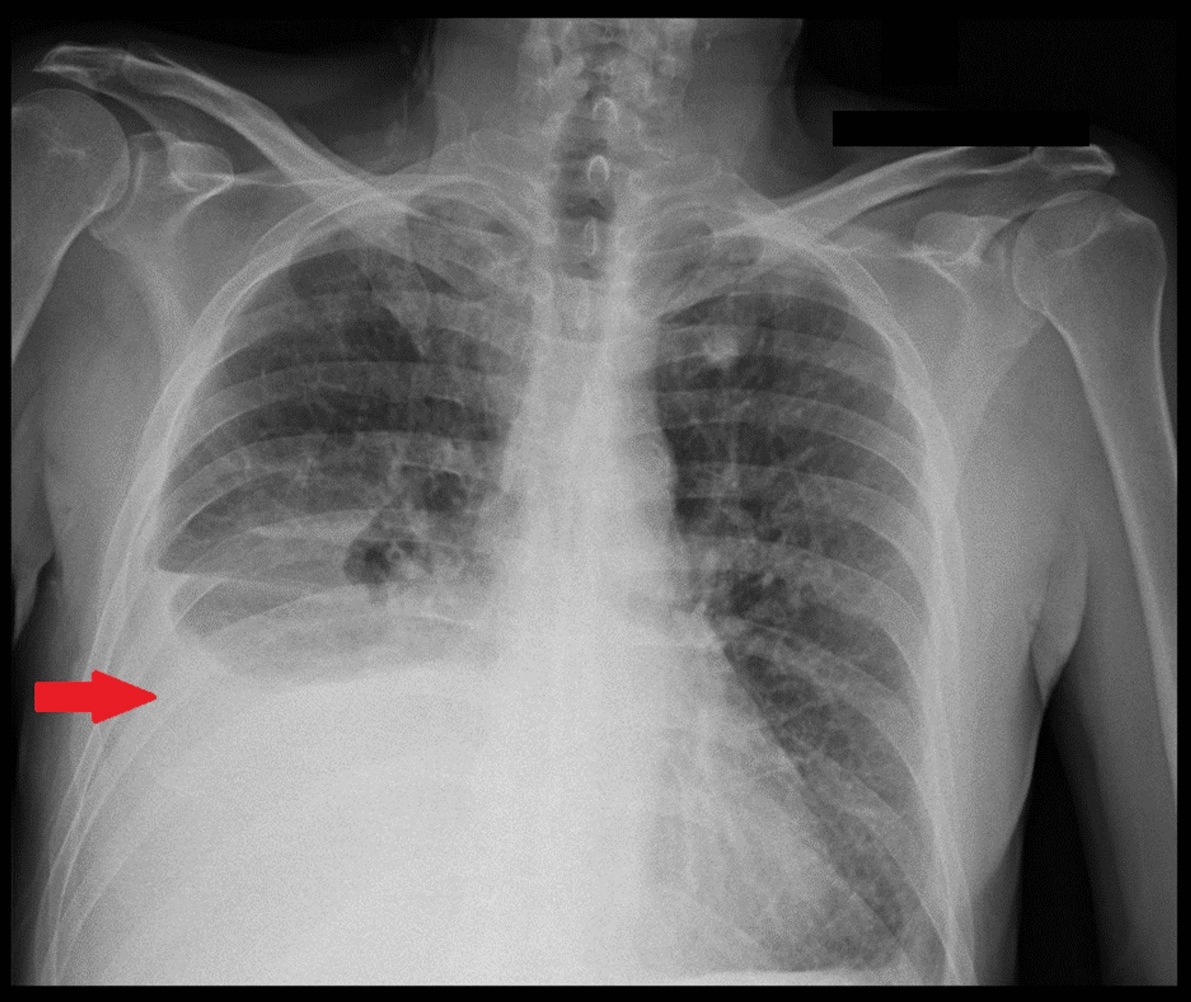
X-ray of the chest showing right pleural effusion with a red arrow with adjacent atelectasis

A 62-year-old male presented with a three-month history of progressive dyspnea, exacerbated by physical activity. His symptom was associated with a persistent productive cough, particularly in the morning, and occasional wheezing. He complained of lower extremity swelling, weight gain, and yellowing of nails. The patient denied hemoptysis, fever, chills, tuberculosis exposure, recent travel, joint pain, skin rashes, dysphagia, visual disturbances, or headaches. His medical history included controlled hypertension, coronary artery disease with stents placed in situ for which he has been on regular follow-up at a cardiology clinic, and skin cancer with surgical resection done 14 years back. He had previously undergone three thoracocentesis procedures. One month prior to this visit, he was admitted for anasarca, and imaging studies showed right pleural effusion (Figure 1). Thoracocentesis was done by draining 800 cc of lymphocytic exudative fluid. Despite the extensive workup, the cause of his edema remained unclear.

**Figure 2:**
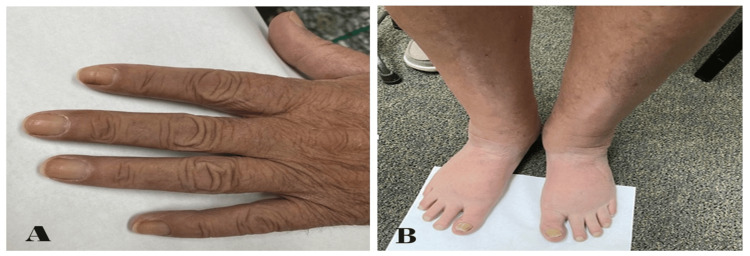
Photographs of the patient's hand and feet: (A) yellowish discoloration of the fingernails of the left hand and (B) mild swelling of bilateral lower limbs with yellowish discoloration of nails

On examination, the patient was an averagely built male, calm, cooperative, and well-oriented to time, place, and person with stable vital signs. He presented with yellow discoloration of the nails of his hands (Figure 2A). On auscultation of the chest, there were decreased breath sounds and dullness on percussion of the left lower side of the chest. In bilateral lower extremities, mild pitting edema was noted with yellow discoloration of nails (Figure 2B). Further examinations of the cardiac, abdominal, and neurological systems revealed no abnormalities.

**Figure 3:**
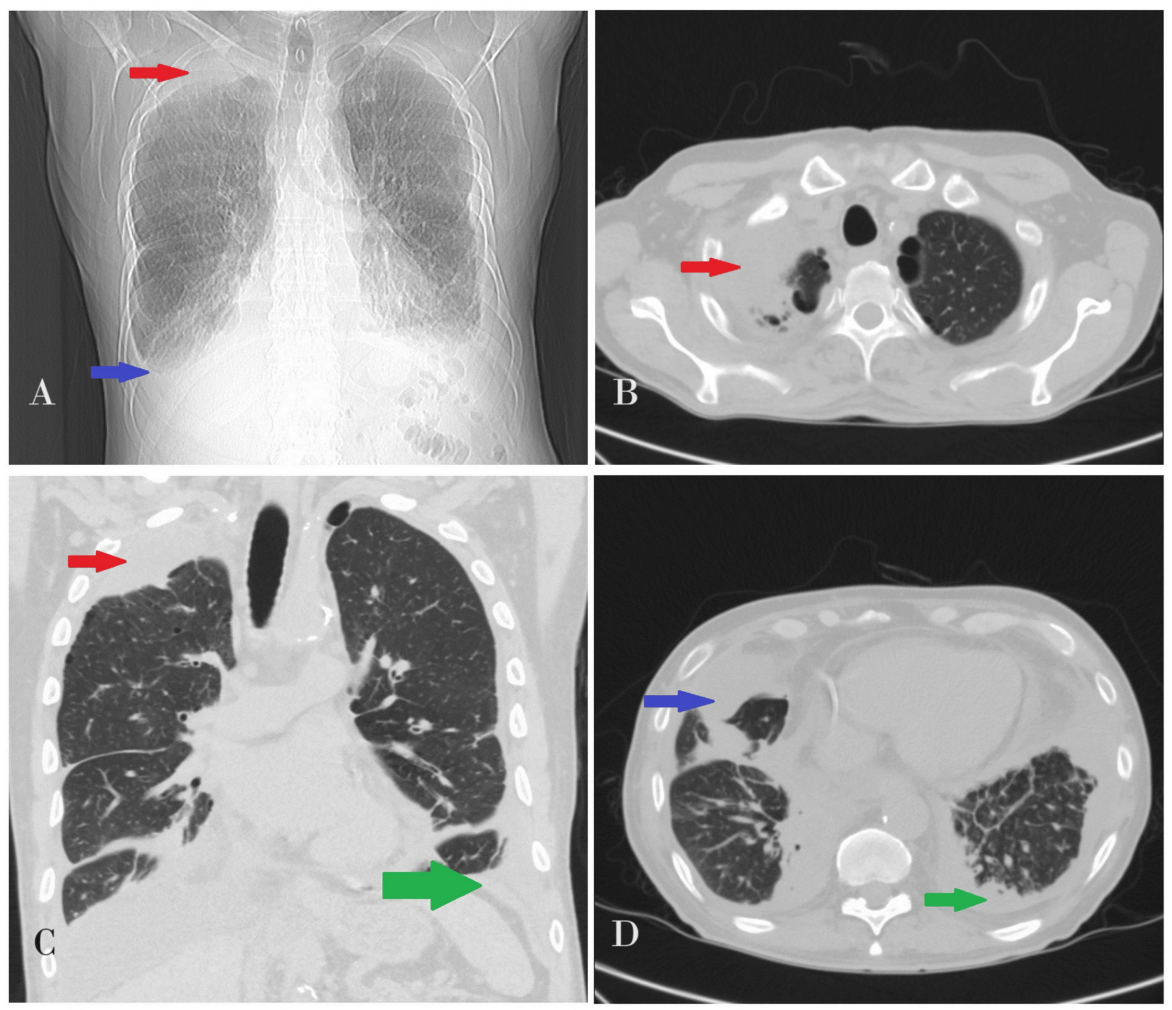
Chest computed tomography scan showing (A and C) pleural effusion in the right apex anteriorly with atelectasis pointed with a red arrow and consolidation in the right lower lobe and left lower pleural effusion with adjacent atelectasis/consolidation pointed with blue and green arrows, respectively; (B) axial view, right-sided partially loculated pleural effusion tracking into the right apex anteriorly pointed with a red arrow; and (D) axial view, atelectasis and consolidation in the right lower lobe and a small left pleural effusion with adjacent atelectasis/consolidation pointed with green and blue arrows, respectively

Initial laboratory investigations, including complete blood count, comprehensive metabolic panel, and blood glucose levels, were all within normal limits (Table 1). Echocardiography revealed normal cardiac structures. Cardiac enzymes and markers showed no signs of cardiac pathology. A computed tomography (CT) scan indicated mild-to-moderate right-sided partially loculated pleural effusion tracking into the right apex anteriorly, as well as adjacent atelectasis and consolidation in the right lower lobe. Additionally, a small left pleural effusion with adjacent atelectasis/consolidation was observed (Figure 3). Subsequently, the patient underwent bronchoscopy with bronchoalveolar lavage, which revealed no endobronchial disease on the left side. Following that, he underwent a left thoracotomy with pleural biopsy and decortication, which showed fibrotic pleuritis with IgG4-positive plasma cells. The patient underwent a rheumatology evaluation to investigate the possibility of IgG4 disease, but his presentation was negative for IgG4-related diseases.

**Table 1:** Laboratory results of the patient

Test	Result	Reference range
White blood cell count	8,750	4,000-10,000/mm^3^
% neutrophils	81.4	40-60%
Red blood cell count	4.27	4.3-5.9 million/mm^3^
Hemoglobin	13.0	13.5-17.5 g/dL
Hematocrit	40.5	41-53%
Platelet	242	150,000-400,000/mm^3^
Serum glucose	107	70-126 mg/dL
Albumin	4.5	3.5-5 gm/dL
Lactate dehydrogenase	188	140-280 U/L
Lactic acid	<0.62	<2 mmol/L
Ammonia	28	15-50 mcg/dL
Procalcitonin	0.11	<0.1 ng/mL
Lipase	20	10-140 U/L
Magnesium	2.5	1.8-2.6 mg/dl
Creatinine kinase	24	30-180 U/L

An extensive evaluation was conducted for suspected causes, which included checking inflammatory markers, infectious causes, autoimmune causes, and malignancies. After excluding all other possible causes and observing the presence of pleural effusion, lymphedema of lower extremities, and yellowish discoloration of the nails in our patient, a diagnosis of yellow nail syndrome was made. Treatment started with a vitamin E supplement for nail changes, compression stockings for leg edema, and pleural fluid drainage for the effusion. The patient showed clinical improvement during regular follow-ups. He was kept under regular observation to evaluate his clinical status. Periodic imaging and laboratory workups were conducted to manage his symptoms and prevent complications.

## Discussion

Yellow nail syndrome is a rare condition, with fewer than 400 cases documented in medical literature. The exact prevalence is unknown, with no regional pattern, and it is estimated to affect less than one individual per 1,000,000 population [1]. Heller et al. first described yellow nail syndrome in 1927 by specifying yellow nail discoloration and lymphedema as the main diagnostic criteria. In 1966, the diagnostic criteria were revised to include pleural effusion and other respiratory tract issues such as chronic cough, recurring pneumonia, recurring sinusitis, bronchiectasis, and pulmonary fibrosis. Again in 1972, Heller et al. suggested that a diagnosis of yellow nail syndrome could be made with only two of the three clinical features [4].

Yellow nail syndrome could be an isolated finding or an initial manifestation of an underlying autoimmune disease or malignancy [1]. The full triad of yellow nail syndrome was referenced in 27-60% of instances [5]. Nail discoloration was characterized by pale yellow to dark green thick nails with increased curvature over time, onycholysis, or sometimes a noticeable hump [1]. It may involve both fingernails and toenails and is essential for diagnosis. The usually reversible nail findings in yellow nail syndrome might be due to the functional nature of the lymphatic obstruction, and such findings tend to improve as the respiratory illness of the patient is better controlled [6].

Pulmonary findings are common, occurring in 56-71% of cases [5]. Chronic cough is the prevailing respiratory symptom and is present in over 50% of the cases [7]. Pleural effusion, either unilateral or bilateral, is also common. Almost 95% of the effusions are lymphocytic-predominant exudates [5]. Pulmonary manifestations can occur anytime during the illness and can range from chronic cough to abnormal X-ray findings, pleural effusions, bronchiectasis, or sinusitis [8]. Impaired lymphatic drainage is suggested by the presence of chylothorax among two-thirds of patients, with the recurring infections of the respiratory tract possibly initiating the pathophysiology involved [6].

In one out of every three cases, non-pitting lymphedema is present [5]. While it is often found on the face and limbs and usually has no response to diuretics which can cause intravascular volume contraction and hence warrants conservative management [3], it is most prevalent on the legs and increases the risk of cellulitis [5]. Bilateral lower limb lymphedema affects 30-80% of cases, leading to complications such as cellulitis and reduced quality of life [7].

Management of yellow nail syndrome involves the utilization of vitamin E, either alone or together with azole antifungal medications, to manage nail discoloration and dystrophy. Oral vitamin E can prevent the formation of lipofuscin pigment, which is thought to cause the discoloration due to its antioxidant properties. Azole antifungal agents may be given for nails, not for their antibacterial properties but for their ability to stimulate linear nail growth [7].

Pulmonary symptoms are managed with the use of antibiotics as prophylaxis for chronic sputum production due to bronchiectasis or surgical management of recurrent pleural effusion with either decortication or pleurodesis, along with chest physiotherapy [1].

Lymphedema is treated with multimodal decongestant therapy, which consists of two stages. The initial step involves using low-stretch bandages, manual lymphatic drainage, and physical exercises to reduce the volume of lymphedema. The second phase focuses on maintaining the volume of lymphedema over time by using compression stockings, physical activity, and proper skincare. This method enhances the quality of life by reducing complications like cellulitis [4,7].

Usually, yellow nail syndrome requires monitoring over time as it may be the initial manifestation of an underlying malignancy, particularly when it has been diagnosed by exclusion [3].

## Conclusions

The cause of yellow nail syndrome is uncertain, and more research is needed to determine its exact origins and potential links to autoimmune diseases, malignancies, or other disease conditions. It may seem harmless and isolated at times, but it could also indicate a potential systemic disease or cancer; therefore, thorough monitoring is crucial for patients.
